# Enhancement of magnetic properties through morphology control of SrFe_12_O_19_ nanocrystallites

**DOI:** 10.1038/s41598-018-25662-8

**Published:** 2018-05-09

**Authors:** Anna Zink Eikeland, Marian Stingaciu, Aref Hasen Mamakhel, Matilde Saura-Múzquiz, Mogens Christensen

**Affiliations:** 1 Center for Materials Crystallography, Department of Chemistry and Interdisciplinary Nanoscience Center (iNANO), Aarhus University, Langelandsgade 140, 8000 Aarhus C, Denmark; 2 Present Address: Physics Department, Institute for Energy Technology, P.O. Box 40, N-2027 Kjeller, Norway

## Abstract

Nanocrystallites of the permanent magnetic material SrFe_12_O_19_ were synthesised using a conventional sol-gel (CSG) and a modified sol-gel (MSG) synthesis route. In the MSG synthesis, crystallite growth takes place in a solid NaCl matrix, resulting in freestanding nanocrystallites, as opposed to the CSG synthesis, where the produced nanocrystals are strongly intergrown. The resulting nanocrystallites from both methods exhibit similar intrinsic magnetic properties, but significantly different morphology and degree of aggregation. The nanocrystallites were compacted into dense pellets using a Spark Plasma Sintering (SPS) press, this allows investigating the influence of crystallite morphology and the alignment of the nanocrystallites on the magnetic performance. A remarkable correlation was observed between the crystallites morphology and their ability to align in the compaction process. Consequently, a significant enhancement of the maximum energy product was obtained after SPS for the MSG prepared sample (22.0 kJ/m^3^), compared to CSG sample, which achieved an energy product of 11.6 kJ/m^3^.

## Introduction

The hard permanent magnetic material strontium hexaferrite, SrFe_12_O_19_ with space group *P*6_3_/*mmc*^1^ has a highly anisotropic crystal structure with unit cell dimensions of *a* = *b* = 5.86 Å and *c* = 23.03 Å^2^. This compound is used in many applications due to its relative high magnetocrystalline anisotropy, non-toxicity, corrosion resistance, mechanical stability and low eddy current losses^3^. It has a theoretical magnetic moment of 20.6 *µ*_B_/unit cell and a Curie temperature around 470 °C^2^. The magnetocrystalline anisotropy constant (*K*_1_) is ~350 kJ/m^3^ along the c-axis of the hexagonal structure resulting in a large theoretical maximum coercivity (*H*_*c*_) of 1592 kA/m, which strongly depends on the crystallite shape and size^2^. The crystallite shape and size is paramount in the optimization of the magnetic performance of SrFe_12_O_19_, and todays commercial SrFe_12_O_19_ have a significantly lower magnetic performance than predicted by theory. The maximum *H*_*c*_ is reached for crystallites having the critical-single-domain size, just before the magnetization of the material divides itself into magnetic multi-domains. The critical-single domain size is estimated to be around 700 nm for SrFe_12_O_19_^4^. However, in this calculation the anisotropic shape of the SrFe_12_O_19_ crystallites is not taken into account. Given the high relevance of the crystallite shape and size regarding the magnetic properties, it is of outmost significance to investigate new methods for controlling the crystallite size and morphology during the synthesis and densification process in order to enhance the magnetic performance. A number of different preparation techniques have been established for preparing SrFe_12_O_19_; solid state reaction method^5,6^, mechanochemical synthesis^7,8^, polyol mediated synthesis^9^, co-precipitation processes^10^, sol-gel synthesis routes^11–15^, and hydrothermal synthesis techniques^15–17^. The sol-gel synthesis route is known to produce SrFe_12_O_19_ nanocrystallites in the magnetic single-domain size range^18^. Furthermore, this technique is characterised by being energy efficient, cheap, fast and reproducible, due to relatively few synthesis steps. The as-prepared powder typically has a *H*_*c*_ of 350 kA/m^13,14,19^, which makes the synthesis technique very promising for producing high-performance SrFe_12_O_19_ magnets. However, one of the main challenges is that the crystallites are partly intergrown into one-another, making them unsusceptible to alignment using pressure or applied magnetic field. Highly aligned crystallites have been reported to significantly improve the magnetic performance of compacted nanopowders^20,21^, and the nanocrystallites ability to freely rotate and align is essential for reaching a high energy product. Sapoletova *et al*. reported a MSG synthesis route, which resulted in plate-like free-standing SrFe_12_O_19_ crystallites with magnetic properties similar to those of as-synthesized SrFe_12_O_19_ crystallites prepared using CSG synthesis^15^.

This paper reports the characterisation of nanocrystallites prepared using both, CSG and MSG synthesis routes. Our hypothesis is that MSG synthesised crystallites can be aligned more easily compared with CSG prepared crystallites, when using high temperature compaction methods like SPS. For the MSG synthesis, we have investigated the nanocrystallite size and morphology as function of the Fe^3+^:Sr^2+^ molar ratio. Furthermore, we have varied the amount of NaCl in the precursor solution to investigate the effect on the synthesized SrFe_12_O_19_ nanocrystallites. Finally, the CSG and MSG synthesised crystallites were compacted using a SPS press in order to investigate how the morphology and aggregation state of the crystallites influences their ability to align when compacted, and how the magnetic properties of the two powder samples with initially similar magnetic properties change after densification.

## Experimental

### Conventional sol-gel synthesis (CSG)

The CSG prepared SrFe_12_O_19_ nanocrystallites were synthesised using the starting materials Sr(NO_3_)_2_ and Fe(NO_3_)_3_∙9H_2_O, citric acid (all Sigma-Aldrich technical grade with purity >98%) and concentrated NH_3(aq)_. The pertinent amounts of citric acid, Sr^2+^-nitrate and Fe^3+^-nitrate were dissolved in a minimum volume of distilled water and mixed by magnetic stirring at room temperature. The Fe^3+^:Sr^2+^ molar ratio was 11.5 since a stoichiometric ratio results in the formation of α-Fe_2_O_3_ impurities^14^. The molar ratio of citric acid to metal cations was 1. Concentrated NH_3(aq)_ was added dropwise to the solution to adjust the pH until it reached pH 7. The solution was dried at 120 °C on a hot plate under constant stirring. The gel was subsequently heated to 250 °C on the hot plate until auto-combustion took place and a grey porous powder was formed. Finally, the powder was calcined in a preheated furnace at 900 °C for 30 minutes.

### Modified sol-gel synthesis (MSG)

The MSG synthesis is a matrix-based synthesis, where the nanocrystallites grow in a solid NaCl matrix. Two different series were prepared in order to investigate the following parameters: 1) the Fe^3+^:Sr^2+^ molar ratio and 2) the amount of matrix material (NaCl) used in the final calcination process. The MSG synthesised SrFe_12_O_19_ nanocrystallites were prepared by dissolving SrCl_2_∙6H_2_O and FeCl_3_∙6H_2_O (≥98% purity, Sigma Aldrich) in distilled water to give a Sr^2+^ concentration of 0.1 M. In the first series, the Fe^3+^:Sr^2+^ molar ratio was varied from 8 to 12 in steps of one. The metal ion solution was then added to a 1 M solution of Na_2_CO_3_ (Chem-Solution GmbH, 99.98% purity) under constant stirring on a hot plate at 90 °C. The synthesis was carried out with 0.5 moles of Na_2_CO_3_ per mole of Cl^-^, resulting in a 1:1 molar ratio between Na^+^ and Cl^-^. After a few minutes of stirring, a 5.5 M solution of citric acid (>99% purity, Sigma Aldrich) was added to the solution. The molar ratio between citric acid and Na_2_CO_3_ was 1.5:1. The mixture was dried to a gel in a convection oven at 120 °C overnight. In the second series of experiments a NaCl solution was added to the mixture before adding the dissolved Na_2_CO_3_. NaCl was added to form a 50%, 100%, or 200% larger matrix than that of the parent study. The Fe^3+^:Sr^2+^ ratio for the NaCl experiments were 9:1. The dried gel was crushed in a mortar and placed in a convection oven at 450 °C for 1 hour to burn off the organic residues. Afterwards, the precursor was calcined at 790 °C for 1 hour, followed by cooling to room temperature at ambient conditions. The melting point of NaCl is 801 °C. Therefore, the nucleation of SrFe_12_O_19_ takes place in a solid NaCl matrix. The product was washed and centrifuged once with 4 M HNO_3_ and four times with distilled water to remove NaCl and SrCO_3_, which is formed due to the excess of Sr^2+^. Finally, the SrFe_12_O_19_ powder was dried in a vacuum oven at 45 °C.

### Powder X-ray diffraction

The as-prepared SrFe_12_O_19_ powders were characterized by powder X-ray diffraction (PXRD) using a Rigaku SmartLab diffractometer equipped with a Co Kα source (Kα_1_ = 1.789 Å, Kα_2_ = 1.792 Å). The data was collected using Bragg-Brentano geometry in the *q*-range 1–5 Å^−1^ (2θ = 16–90°). The diffracted X-rays were collected using a D/teX Ultra detector. Rietveld refinements were carried out using the FullProf Suite software package^22^. Impurities were identified and the crystallite size and unit cell parameters were determined from the Rietveld analysis. A NIST 660B LaB_6_ standard measured under identical conditions was used to correct for instrumental broadening.

### Spark Plasma Sintering

The synthesized SrFe_12_O_19_ powders were compacted into pellets using a Spark Plasma Sintering (SPS) press - SPS Syntex Inc., Dr. Sinter Lab^TM^ series. The pellets were produced by loading ~0.4 g of powder sample into a graphite matrix of 8 mm inner diameter. The resulting pellets have a typical thickness of ~1 mm after polishing off the graphite paper. Three pellets were made from the MSG synthesised powder at a pressure of 100 MPa and at temperatures of 750, 800 or 850 °C with a holding time of 2 minutes. One pellet was made from CSG powder at 100 MPa at 850 °C and held for 2 minutes. The measured densities, obtained by the geometry and weight of the pellets, were 97% and 92% for MSG and CSG pellets, respectively, compared with the theoretical density.

### Pole figure measurement

The previously mentioned Rigaku Smartlab diffractometer was also used for pole figure measurements. Here, cross beam focus optics (CBO-f) was used to produce an approximate 0.4 × 0.4 mm^2^ sized beam. The reflections (110), (008), (107) and (114) were selected for pole figure measurements, and the background was collected at 2θ = 61.2°. The pole figures were obtained by performing 360° ϕ-scans in 5° steps and χ scans between 75° and 0°, likewise in 5° steps. The obtained pole figures were evaluated using the MTEX software version 3.4.1 to extract the orientation distribution function (ODF)^23^.

### Magnetisation measurements

A Physical Property Measurement System (PPMS) from Quantum Design, equipped with a Vibrating Sample Magnetometer (VSM) was used for assessing the magnetic properties of the powders as well as the pellets. The as-prepared powders were cold pressed into pellets with 3 mm diameter and ~1 mm thickness, while the SPS pressed pellets were cut into rectangular shapes with approximate dimensions of 2 × 2 × 1 mm^3^. Cylindrical near zero background brass holders were used together with quartz rods to hold the sample in place during the measurement. Hysteresis curves were measured at 300 K in an external magnetic field varied between ±2393 kA/m (±3 T). The measured hysteresis curves in case of loosely compacted powder was correct by a demagnetizing factor of *N* = 0.33, were as the SPS pellets were correct using the graphical infinite slope method reported by Saura-Múzquiz *et al*.^20^. (see supporting information for more details, Fig. S1). In all cases a sample density of 5.1 g/cm^3^ is assumed to allow comparison between loosely packed and SPS compacted samples.

### Transmission electron microscopy

The as-prepared powders were suspended in ethanol using an ultrasonic bath before being transferred to a copper grid sample holder with a carbon thin film layer. Images of the nanoparticles were obtained using transmission electron microscopy (TEM), collected on a TALOS F200A (200 kV) with a TWIN lens system, X-FEG electron source and Ceta 16 M Camera.

## Results and Discussion

### Sol-gel synthesised SrFe12O19

**Figure 1:**
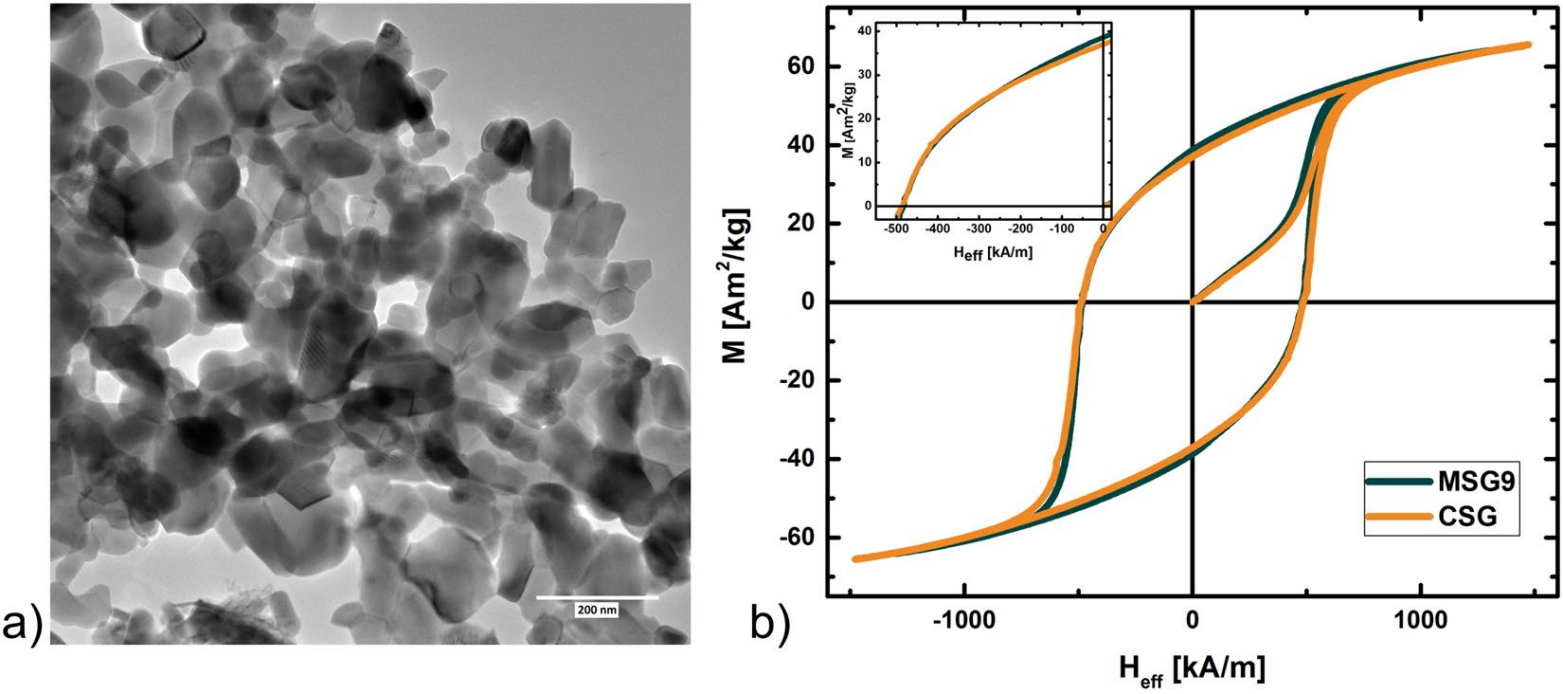
(**a**) TEM image of CSG synthesised SrFe_12_O_19_. The scale is 200 nm. (**b**) Hysteresis curves of the powder samples MSG9 and CSG.

Phase-pure SrFe_12_O_19_ was synthesised using the CSG technique. From Rietveld refinement of the powder diffraction data the *a* = *b* unit cell parameter is found to be 5.88077(2) Å and the *c*-axis is 23.0607(1) Å. The crystallites were refined as anisotropic platelets with the platelet normal parallel to the crystallographic *c*-axis. Therefore, two sizes were extracted; one along the *ab*-axes (AB-size) and one along the *c*-axis (C-size). The refined crystallite sizes are 70(1) and 62(1) nm for the AB-size and C-size, respectively. In other words, the shape is almost isotropic with an AB/C aspect ratio of 1.13. The morphology of the CSG synthesised particles was investigated by transmission electron microscopy (TEM) images; as illustrated on Fig. 1a). The particles are intergrown, making it difficult to determine the morphology. The sizes are estimated to be between 20–150 nm, in good agreement with the average size obtained from the Rietveld refinement. The sizes are also in agreement with previously published papers on CSG synthesised SrFe_12_O_19_, which also report highly aggregated crystallites^12–14^. An elemental analysis of the sample along with powder diffraction patterns can be found in supporting information Figs S2, S3.

For comparison the hysteresis curve of the CSG synthesised powder sample is plotted in Fig. 1b) together with the MSG9 powder sample. The coercivity, remanence and magnetization values are practically identical for both samples: *M*_*s*_ at applied field of 1500 kA/m is 63.8 Am^2^/kg. The remanence *M*_*r*_ is 37.0 Am^2^/kg, and the coercivity *H*_*c*_ is 487 kA/m and the resulting *BH*_*max*_ reaches 9.4 kJ/m^3^ using a demagnetising correction *N* = 0.33 and using theoretical density of 5.1 g/cm^3^ details are given in supporting information.

### MSG Fe3+:Sr2+ molar ratio study

**Figure 2:**
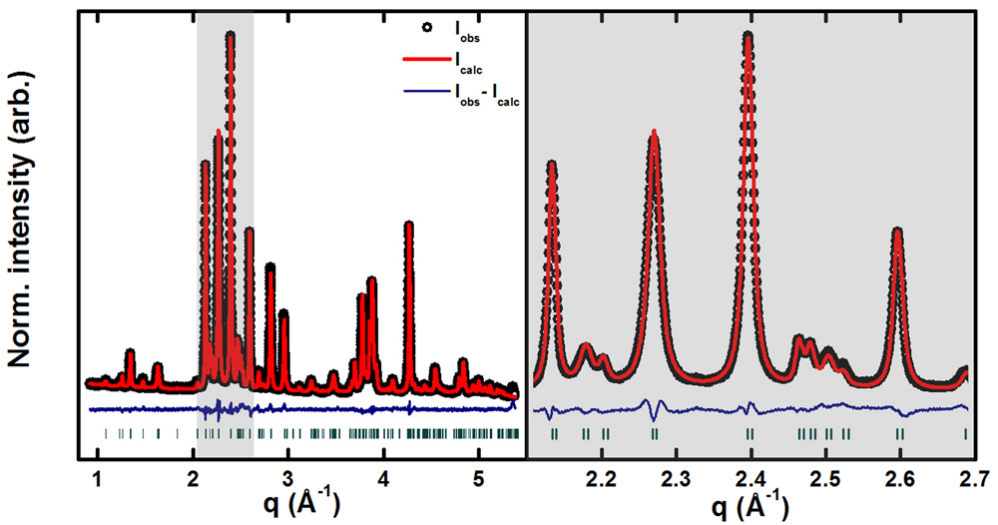
PXRD patterns and Rietveld refinements of SrFe_12_O_19_ nanocrystallites prepared from the Fe^3+^:Sr^2+^ molar ratio 9 (MSG9). To the right a zoom of the data between 2.1 and 2.7 Å^−1^ is shown.

In the MSG synthesis, the molar ratio of Fe^3+^ and Sr^2+^ was varied from 8 to 12 and the samples are named MSG8, MSG9, MSG10, MSG11, and MSG12 according to the Fe^3+^:Sr^2+^ molar ratio. The collected PXRD patterns of all samples are shown in (supporting material Fig. S4), initially prepared MSG11 and MSG12 had impurities of α-Fe_2_O_3_, however it was later hypothesised, that access to oxygen in the second synthesis step is essential for avoiding formation of α-Fe_2_O_3_ (more details in supporting information, Fig. S5). More investigations would be necessary to prove the hypothesis, but this is outside the scope of this paper. The powder diffraction pattern for MSG9 is shown as an example in Fig. 2: black dots are the collected data, the red line is the calculated intensity extracted from the Rietveld refinement, and the blue line is the difference between the observed and calculated intensities. The green lines are the Bragg peaks positions corresponding to SrFe_12_O_19_. All samples are phase pure SrFe_12_O_19_ as can be seen in supporting material. PXRD patterns of the non-reacted gel, the intermediate product heated at 450 °C and the final product is likewise provided in the supporting material Fig. S6.

**Figure 3:**
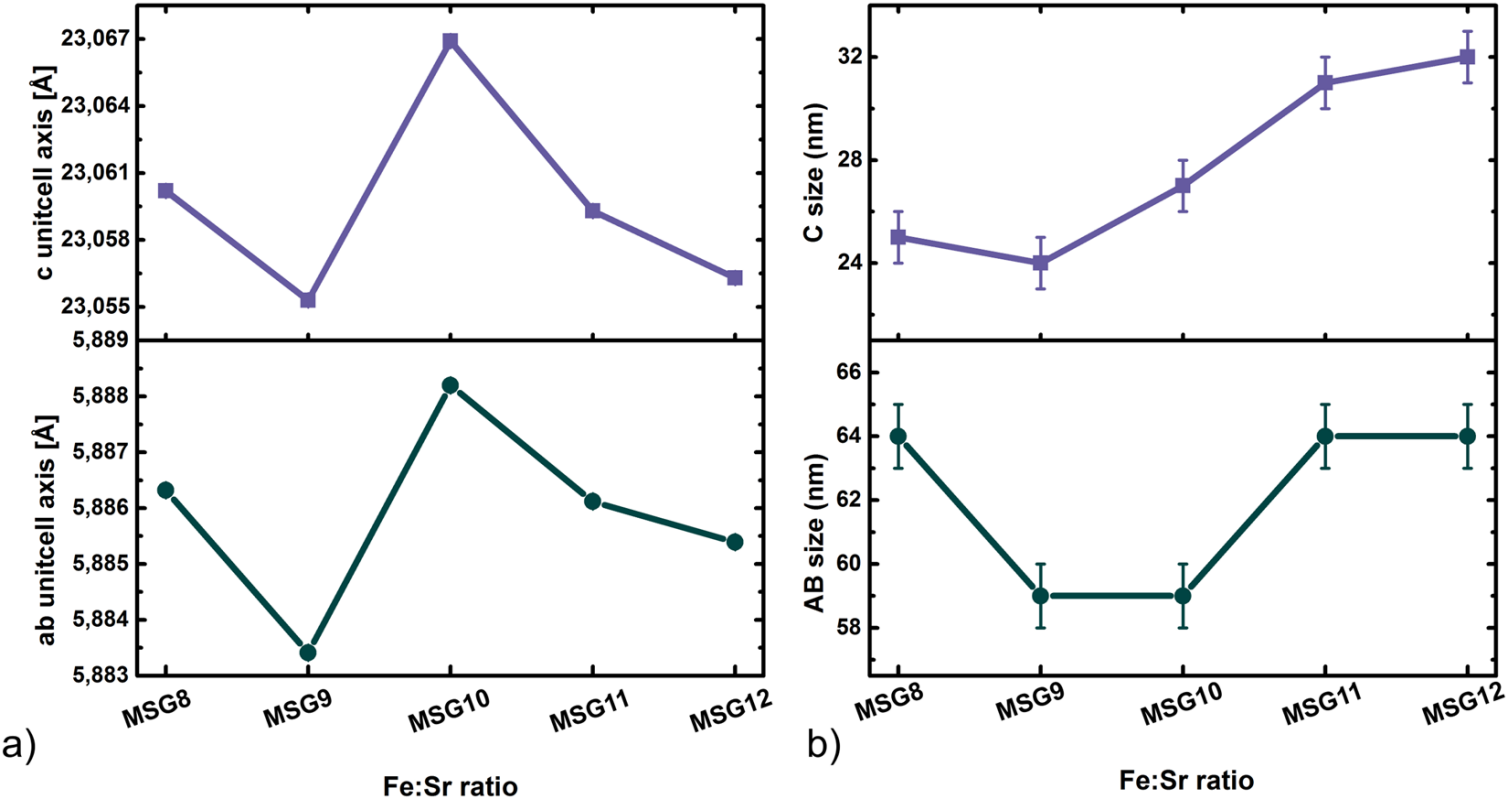
(**a**) Unit cell axis for SrFe_12_O_19_ prepared by MSG at different Fe^3+^:Sr^2+^ molar ratios ( = 8–12). (**b**) Crystallite sizes for SrFe_12_O_19_ prepared by MSG at Fe^3+^:Sr^2+^ ratios from 8 to 12 in steps of one.

Within the resolution of the experimental data all samples are almost identical with respect to the unit cell parameters. The *ab*-axis varies from 5.88341(2) Å (MSG9) to 5.88820(2) Å (MSG10), and similar variations are observed for the *c*-axis (See Fig. 3a). The absolute uncertainty on the extracted unit cell parameters are expected to be in the order of ±0.02% of the determined unit cell value. The variations are small and considered to be below the absolute uncertainty associated with the refinements. The crystallite sizes obtained from Rietveld refinements are plotted in Fig. 3b). The size varies from 59 to 64 nm for the AB-size and from 24 to 32 nm for the C-size, giving an AB/C ratio in the range of 2–2.5. The C-size appears to be slightly affected by a change in the Fe^3+^:Sr^2+^ molar ratio, where increasing molar ratio increases the C-size. No trend in crystallite size is observed along the AB-size when changing the Fe^3+^:Sr^2+^ molar ratio. These results are contrary to the synthesis of SrFe_12_O_19_ using the sol-gel synthesis method^24^, hydrothermal synthesis^25^, and microwave-induced combustion processes^26^ where the crystallite increases significantly with increasing Fe^3+^:Sr^2+^ molar ratio.

**Figure 4:**
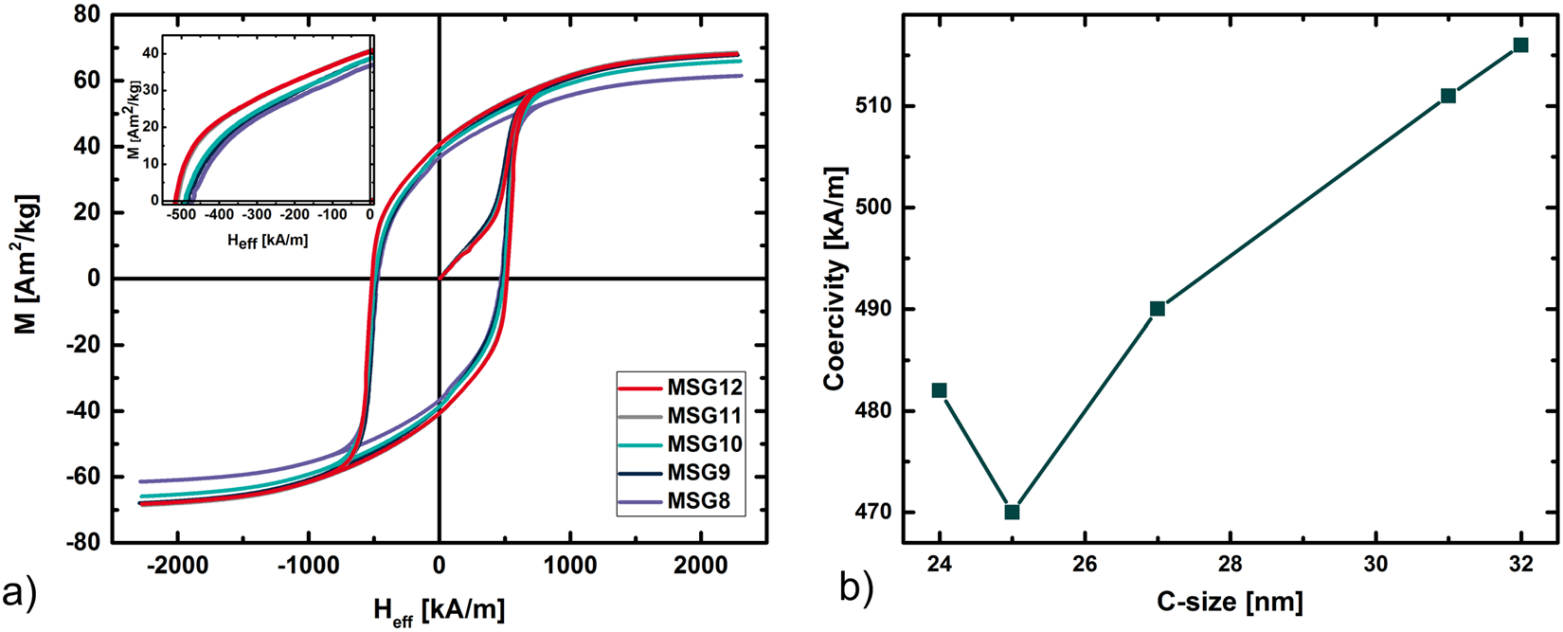
(**a**) Hysteresis curves (magnetisation *vs* effective field) of SrFe_12_O_19_ synthesised by MSG, having the Fe^3+^:Sr^2+^ molar ratios varied from 8 to 12. The demagnetising factor *N* was estimated to 0.33 as the particle shape is roughly approximated to be a sphere. (**b**) The coercivity generally increases as the crystallite C-size is increased.

The magnetic hysteresis loops given in Fig. 4a) show only slight variations between samples. The sample having the best saturation magnetisation, *M*_*s*_, is MSG11 (=68.5 Am^2^/kg) whereas MSG8 has the lowest *M*_*s*_ (=61.5 Am^2^/kg). The coercivity varies from 470 to 516 kA/m for MSG8 and MSG11, respectively. It seems that the coercivity is increased by increasing Fe^3+^:Sr^2+^ molar ratio (Fig. 4b). This is most likely a consequence of an increased shape anisotropy caused by the longer C-size along the easy magnetization axis of the material.

The following studies are made using MSG9, partly because it has the smallest dimensions along AB- and C-sizes, and because the initially prepared MSG11 and MSG12 had impurities of α-Fe_2_O_3_. Later, it was realized that oxygen access to the entire sample is essential for SrFe_12_O_19_ formation, and too thick layers of material during the calcination causes the formation of α-Fe_2_O_3_. The similar crystallite sizes and relatively small differences in the magnetic properties suggest that the choice of the starting powder will be of minor importance for the study of the compaction process.

### MSG prepared with an excess of NaCl

**Figure 5:**
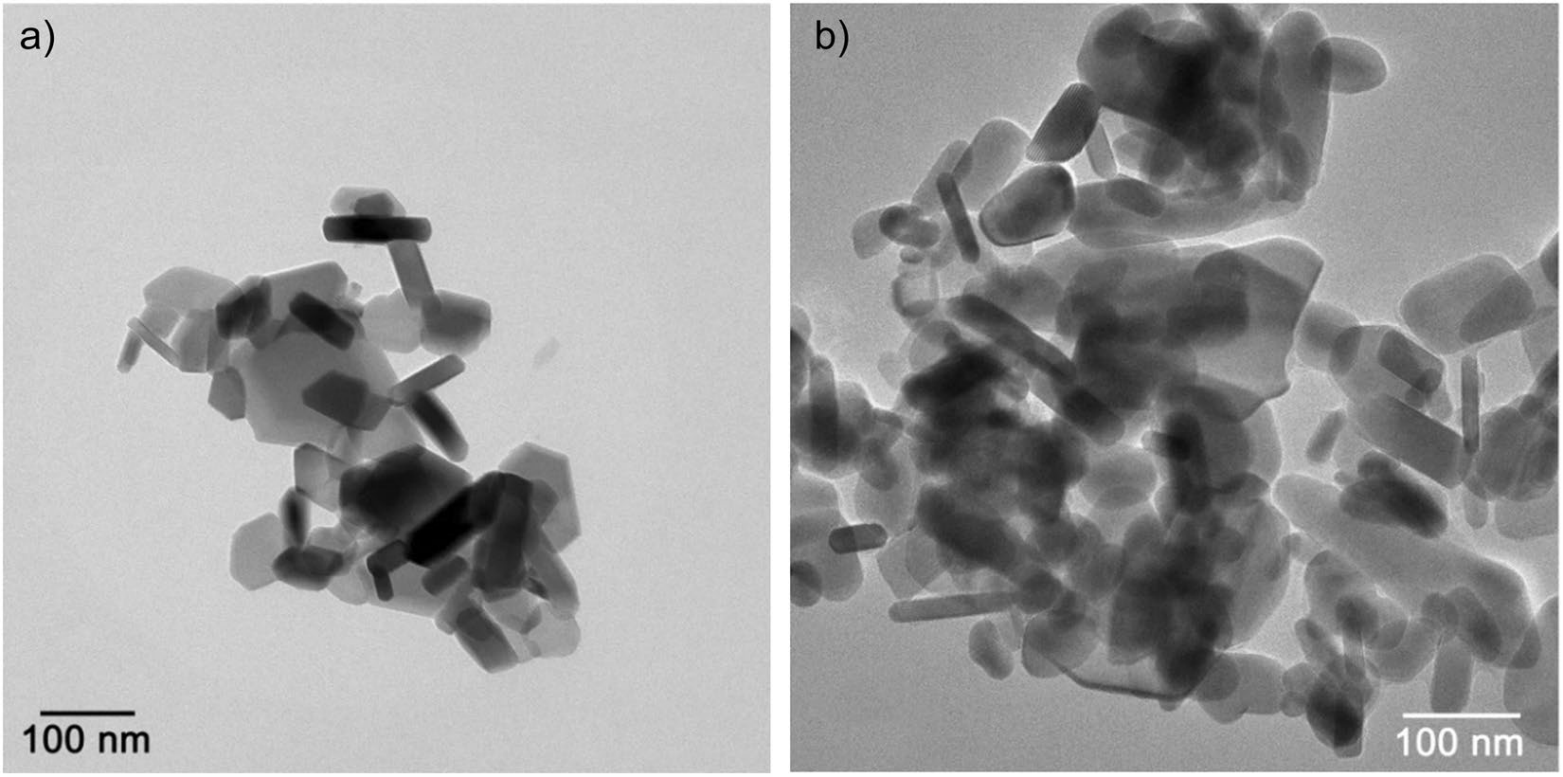
(**a**) TEM image of MSG synthesised SrFe_12_O_19_ with the Fe^3+^:Sr^2+^ molar ratio 9 (MSG9). (**b**) TEM image of MSG300 where 300% extra NaCl was added to the precursor.

**Figure 6:**
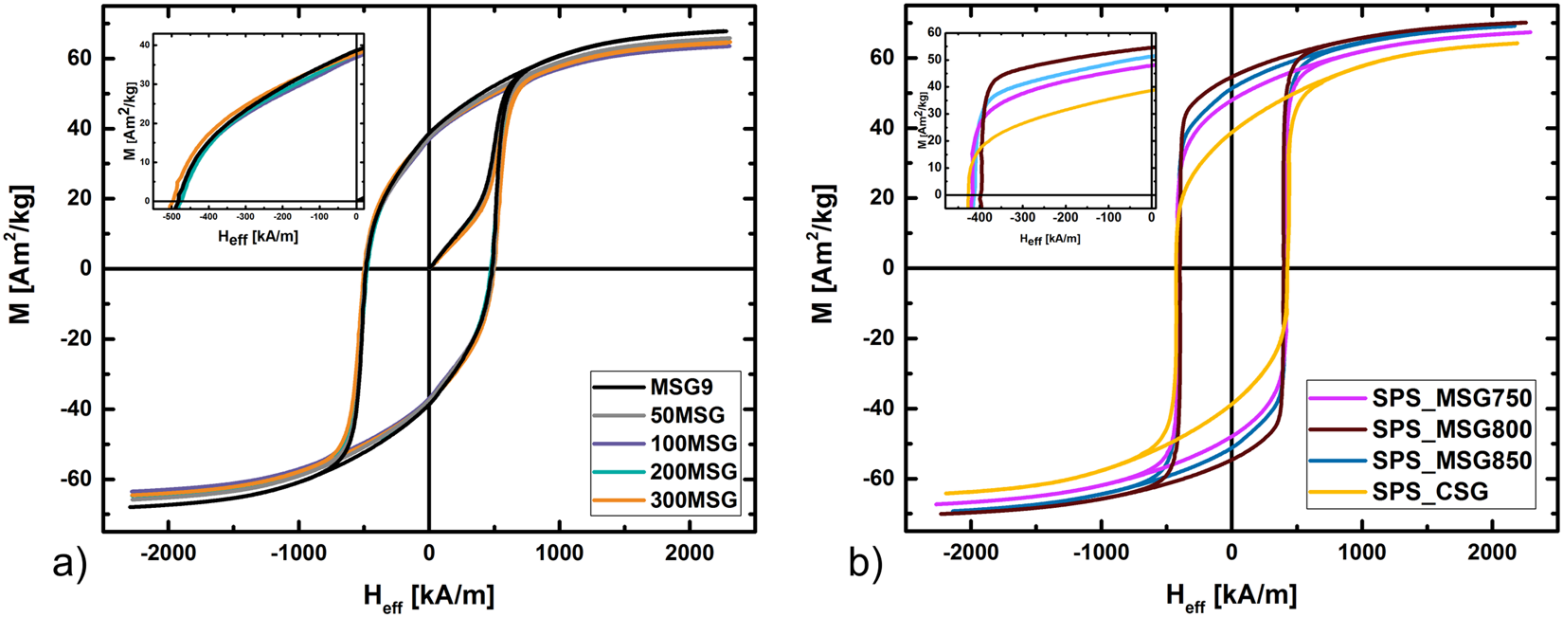
(**a**) Hysteresis curves of MSG samples added between 50 and 300% extra NaCl relative to the precursor. (**b**) Hysteresis curves SPS compacted pellets made from CSG or MSG synthesised powder.

The samples synthesized with an excess of NaCl to further disperse the SrFe_12_O_19_ are denoted as 50MSG, 100MSG, 200MSG and 300MSG, depending on the percentage of NaCl added to the precursor with respect to the NaCl formed by the reaction between the metal-chlorides and Na_2_CO_3_. The molar ratio Fe^3+^:Sr^2+^ used for the NaCl study was 9. From X-ray powder diffraction, all products are almost phase pure SrFe_12_O_19_, except for a very small amount (<0.5 wt%) of α-Fe_2_O_3_ impurity, which is only detectable due to the very high data quality obtained by using the Co X-ray source. The *a* = *b* unit cell axis varies from 5.88615(2) Å to 5.88722(2) Å for the samples with 300MSG and 200MSG (see supporting information Figs S7, S8). The smallest refined *c*-axis belongs to MSG100 (=23.05758(3) Å), while the largest *c*-axis is refined for 200MSG (=23.0631(2) Å). The variations in unit cell axes are not significant and it is reasonable to believe that an excess of NaCl does not affect the unit cell parameters. The variation is rather giving the absolute precision of the refinement. The AB-size varies from 56(1) to 60(1) nm for 50MSG and 300MSG, respectively. The C-size is identical within uncertainties with a size of 25(1) nm. Based on the Rietveld refinements it is concluded that the addition of NaCl does not change the morphology noticeably. A minor increase of the AB-size is observed, but the variations are small and non-systematic, therefore it is concluded based on powder diffraction data that the crystallite morphology is unchanged. A slightly different picture is revealed by transmission electron microscopy (TEM). The TEM images of MSG9 and 300MSG are shown in Fig. 5a,b). The particles of MSG9 have an AB-size between 20 and 150 nm and a C-size between 20 and 30 nm, which is in good agreement with values found from PXRD. 300MSG particles are within the same size range as those of MSG9, but MSG9 clearly have a hexagonal shape, contrary to 300MSG where the crystallites have more rounded corners. An elemental analysis of the MSG9 synthesised powder can be found in Fig. S9 in supporting information. In conclusion, the TEM and powder diffraction sizes are in agreement likewise the platelet morphology and the hexagonal structure can be seen for the particles, which are positioned with the *c*-axis parallel to the electron beam. The magnetic performance of the samples with different added content of NaCl is very similar as can be seen from Fig. 6a). *M*_*s*_ varies from 63.5 to 65.9 Am^2^/kg, the magnetic remanence, M_r_, varies from 36.9 to 37.8 Am^2^/kg, and the *H*_*c*_ of the samples are in the interval between 475 and 489 kA/m. Consequently, the energy product, *BH*_*max*_, for the four samples made with excess of NaCl is similar and with values between 9 to 10 kJ/m^3^, which is comparable to the as-prepared SrFe_12_O_19_ without additional NaCl. Diffraction, TEM images and magnetometry results confirm the similarity between the samples prepared with and without addition of NaCl in the MSG synthesis of SrFe_12_O_19_.

### SPS compacted samples

**Figure 7:**
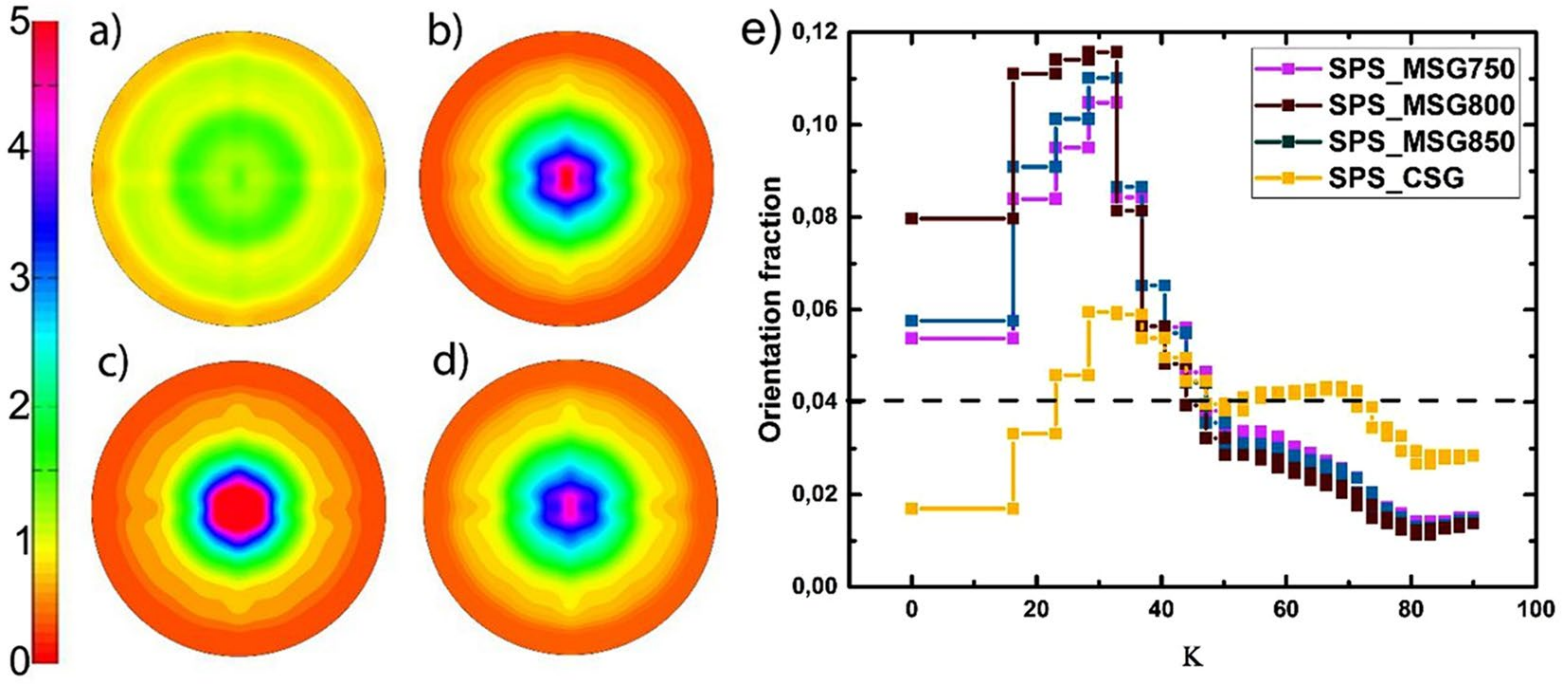
Orientation distribution function (ODF) of (00*l*) poles obtained from X-ray PF measurements of (**a**) SPS_CSG and (**b**) SPS_MSG850, (**c**) SPS_MSG800, (**d**) SPS_MSG750, (**e**) Oriented volume fraction of SPS_MSG750, SPS_MSG800, SPS_MSG850, and SPS_CSG with increasing Κ angle, obtained from the extracted ODF.

Four pellets were obtained by SPS compaction of synthesized powders. Three pellets were made from MSG9 synthesised powder and pressed at temperatures of 750, 800 and 850 °C. The pellets are called SPS_MSG750, SPS_MSG800, and SPS_MSG850 depending on the compacting temperature. The pellet made from CSG synthesis method was compacted at 850 °C and is called SPS_CSG. The orientation distribution function (ODF) of the SPS pellets was obtained from the X-ray pole figures, using the MTEX software. The resulting (*00l*) pole figure is shown for the four SPS pellets in Fig. 7. The obtained raw data can be found in supporting information Figs S10, S11. The orientation distribution observed on the (*00l*) pole figure of sample SPS_CSG is practically homogeneous throughout the entire pole, indicating a close to random orientation of the crystallites in this sample (Fig. 7a). On the other hand, the pole figures of the SPS_MSG samples all show a higher degree of crystallite alignment, indicating alignment of the MSG powder during compaction. From the ODF it appears that the SPS_MSG800 has a slightly higher degree of alignment compared with the pellets pressed at 750 (SPS_MSG700) and 850 °C (SPS_MSG850). However, the alignment of the SPS_MSG pellets is far from perfect. In Fig. 7e) the volume fraction distribution from the extracted ODF with increasing *Κ* is plotted. The steps in *Κ* are made in such a way, that they present a constant integrated volume of the orientation hemisphere, when going from the pole to the equator. A value of 0.04, shown in Fig. 7e) as a dashed line, corresponds to a random distribution.

The extracted orientation volume fraction for the SPS_MSG pellets show that ~54–60% of the crystallites are oriented within the first 40°, where SPS_MSG800 has the largest degree of alignment and SPS_MSG750 has the lowest. For SPS_CSG the values in the different volume fractions vary around the random orientation with ±3%. The data reveal the SPS_MSG pellets to be significantly better aligned than SPS_CSG. Previous studies of SPS compaction have been reported for pellets made from SrFe_12_O_19_ platelet crystallites synthesised by hydrothermal methods^21,27^. In both studies, the powder samples used for SPS compaction are characterised by being hexagonal platelet-like particles with large AB/C aspect ratios. The TEM images of those studies show AB/C aspect ratios approaching 10 and 100 for flow and autoclave synthesis, respectively^20,21^. These are significantly larger than those observed here, where the aspect ratio from TEM approaches 1 and 5 for the CSG and MSG, respectively. The obtained volume orientation fraction for the hydrothermal flow synthesised samples showed highly aligned SPS pellets, with ~50% of the crystallites oriented within 25° of the *00l*-direction^20^. The pellets made from hydrothermal autoclave crystallites revealed an orientation 58% of the volume aligned within 25° of the (*00l*) direction^21^. The ability of the crystallites to align by SPS is size and shape dependent: Isotropic and intergrown crystallites, like those of CSG, show less alignment than plate-like, freestanding crystallites. The previously reported studies also suggest that small plate-like crystallites with small AB/C aspect ratios do not align as easily as large plates with large AB/C aspect ratios. The MSG investigations further support this observation.

### Magnetic properties of SPS samples

The magnetic performance of the four SPS compacted pellets, plotted in Fig. 6b), varies remarkably from SPS_CSG. In case of SPS_CSG there is only a small variation in the *M*_*r*_/*M*_*s*_ value compared to that of the starting CSG powder, the ratio increasing from 0.53 for the powder to 0.60 for the pellet. For the SPS_MSG pellets, the *M*_*r*_/*M*_*s*_ value is increased by 20–27% with respect to the original powders, from 0.57 to 0.71–0.78, depending on the compacting temperature. This indicates that the crystallites become more aligned when compacted using the SPS press compared with the as prepared sample at room temperature. For all pellets, *H*_*c*_ decreases compared with the starting powder. SPS_CSG decreases by 12% to 427 kA/m, resulting in the best *H*_*c*_ for the compacted pellets. The *H*_*c*_ of the SPS_MSG pellets drops by 13–17%, compared to the initial powders, resulting in 417 kA/m for SPS_MSG750 and 398 kA/m for SPS_MSG800. SPS_CSG has a *BH*_*max*_ of 10.8 kJ/m^3^, which is only 13% larger than the as-synthesised powder, whereas the maximum energy product is improved by >100% when the MSG synthesised powder is compacted to a pellet by SPS, leading to a *BH*_*max*_ of 22.0 kJ/m^3^. Furthermore, the crystallites made from MSG synthesis are more dense (97% of the theoretical density) compared to 92% density for CSG compacted powder.

The previously reported pellets made from SrFe_12_O_19_ crystallites synthesised using autoclaves have a higher *BH*_*max*_ due to the high degree of alignment^20,21^. However, the *H*_*c*_ for SPS_MSG is more than double of the autoclave SPS pellets, from 193 to 417 kA/m, which is around 100 kA/m higher than the conventional commercial SrFe_12_O_19_ available today^28^.

The MSG synthesis route tends to partly solve one of the big challenges associated with using high coercivity SrFe_12_O_19_ obtained by sol-gel synthesis techniques, where the intergrown nanocrystallites prevents efficient alignment, which is a fundamental necessity for anisotropic magnets in order to take advantage of the full crystallite ensemble. The MSG prepared powder reported here, with saturation magnetisation extracted at 2000 kA/m *M*_*s*_ = 68.5 Am^2^/kg (MSG11), has similar magnetic properties compared to previously reported MSG powders by Sapoletova *et al*. (*M*_*s*_ = 72.0 Am^2^/kg)^15^. Sapoletova *et al*. reported a *H*_*c*_ of 509 kA/m, similar to MSG12 having *H*_*c*_ of 516 kA/m. The MSG prepared crystallites do not align as much as samples prepared by hydrothermal synthesis methods. However, the alignment may be further improved by, for example by, applying an external magnetic field to the sample prior to compaction^21^. This needs to be further investigated.

## Conclusion

Phase pure SrFe_12_O_19_ was prepared using the well-known conventional sol-gel method (CSG) and a modified sol-gel synthesis method (MSG) using a NaCl matrix during crystallite growth to obtain freestanding nanocrystallites. The crystallites produced using the MSG technique is easily reproducible; their morphology and magnetic properties do not vary considerably when changing the Fe^3+^:Sr^2+^ molar ratio or when adding an excess of NaCl to the precursor. The obtained crystallite size is around 60 nm along the AB direction and 25 nm along the C axis. MSG11 got the best *M*_*s*_ at 2000 kA/m, equal to 68.5 Am^2^/kg, whereas MSG12 has the best *H*_*c*_ = 516 kA/m resulting in the largest powder *BH*_*max*_ of 11.33 kJ/m^3^. The magnetic properties of the CSG synthesised powder sample were slightly worse than the MSG synthesised sample, with *M*_*s*_ and *H*_*c*_ equal to 63.8 Am^2^/kg and 487 kA/m, respectively.

In general, the particles prepared using the MSG technique are non-agglomerated and have the characteristic hexagonal platelet-like shape of SrFe_12_O_19_. In comparison, the CSG particles are highly agglomerated and partly intergrown. The different morphologies and the agglomeration state of the samples result in a remarkably different behaviour when the powders are compacted by SPS. Obtained pole figure measurements verify that the SPS_MSG nanocrystallites are significantly more aligned, independently of the compacting temperature, than the nanocrystallites in the SPS_CSG pellet. Consequently, the energy product was increased by >100% after SPS compaction of the as-prepared MSG powders, from 10.3 kJ/m^3^ to 22.0 kJ/m^3^, whereas the energy product for the CSG powder sample is almost unchanged after compaction by SPS.

## Electronic supplementary material

Supporting information
