# Supplementary material for: Enhancement of magnetic properties through morphology control of SrFe12O19 nanocrystallites
Source: Sci Rep. 2018 May 9;8:7325. doi: 10.1038/s41598-018-25662-8 (PMC5943258; doi:10.1038/s41598-018-25662-8)
Supplement: Supplementary file 1 — Supporting information [file 41598_2018_25662_MOESM1_ESM.pdf]

Supporting information:

Enhancement of magnetic properties through morphology control of $SrFe_{12}O_{19}$ nanocrystallites

Anna Zink Eikeland, Marian Stingaciu, Aref Hasen Mamakhel, Matilde Saura-Múzquiz and Mogens Christensen

The demagnetization factor

In the vibrating sample magnetometry (VSM) the sample is magnetised in an open magnetic field configuration. It is necessary to correct for the sample demagnetising factor, *N*, to obtained the effective magnetic field H_{eff} using the applied field, H_{app}, and the magnetisation of the sample, *M*; $H_{eff} = H_{app} - NM$. The demagnetising factor *N* is affected by macroscopic shape and microscopic effects including: the crystallite shape, size and size distribution as well as orientation distribution, also have an effect. Therefore, it is very challenging and often unpractical to determine the exact *N*. Consequently, *N* was determined graphically as reported by Saura-Múzquiz *et al*,[21](in supporting information) so that the hysteresis curves show an infinite slope at H_c.

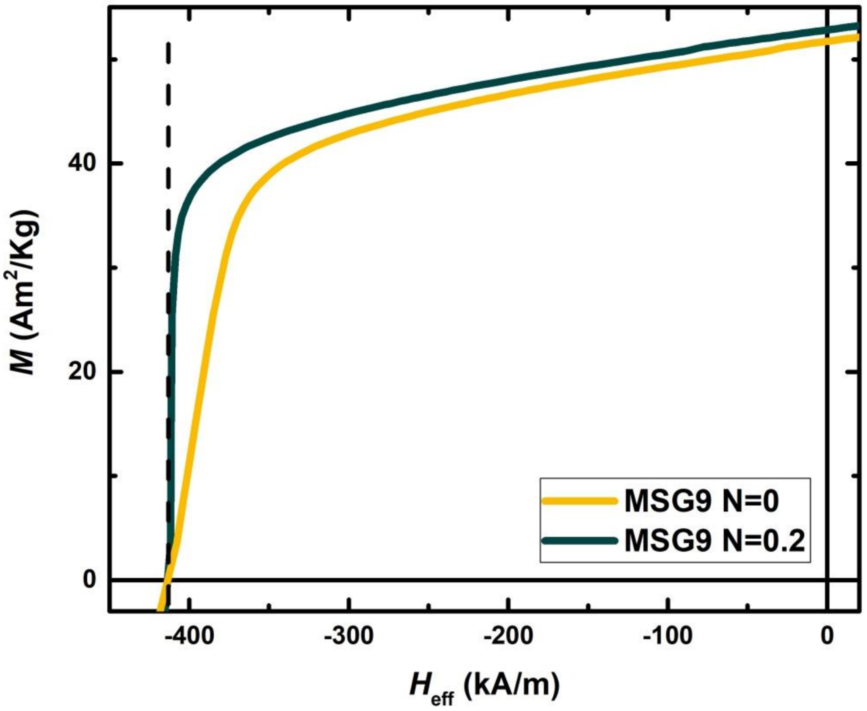

Figure S1: The 2nd quadrant of the hysteresis curve for the uncorrected (yellow) and the corrected (dark green) modified sol-gel sample after SPS compaction. The dashed line is a guide to the eye to emphasis the 'infinite' slope at the coercivity.

Additional figures

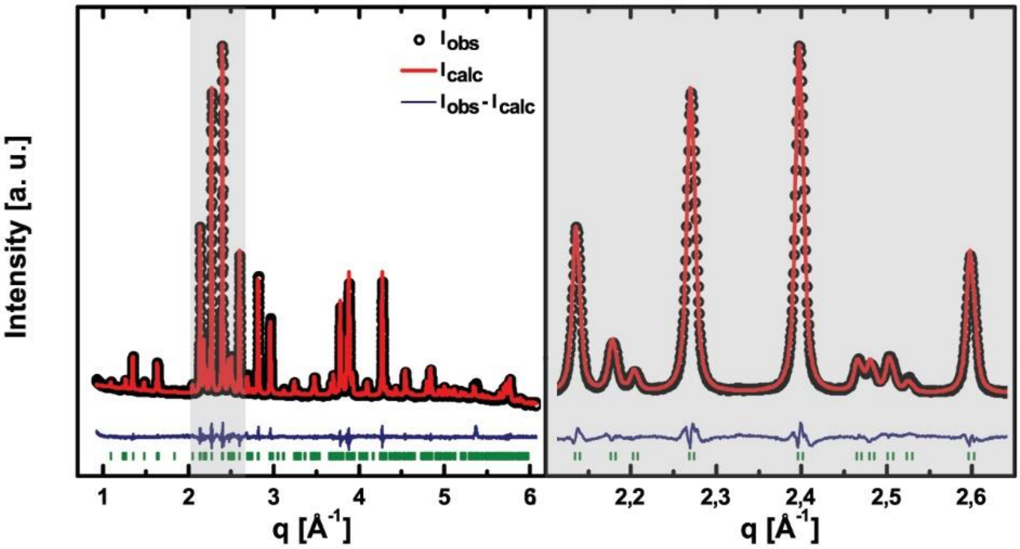

Figure S2 PXRD and Rietveld refinement of conventional sol-gel synthesized powder. The black dots are the observed intensities, the red line is the refined model, the blue line is the difference between the two, and the green lines are Bragg peaks of $SrFe_{12}O_{19}$.

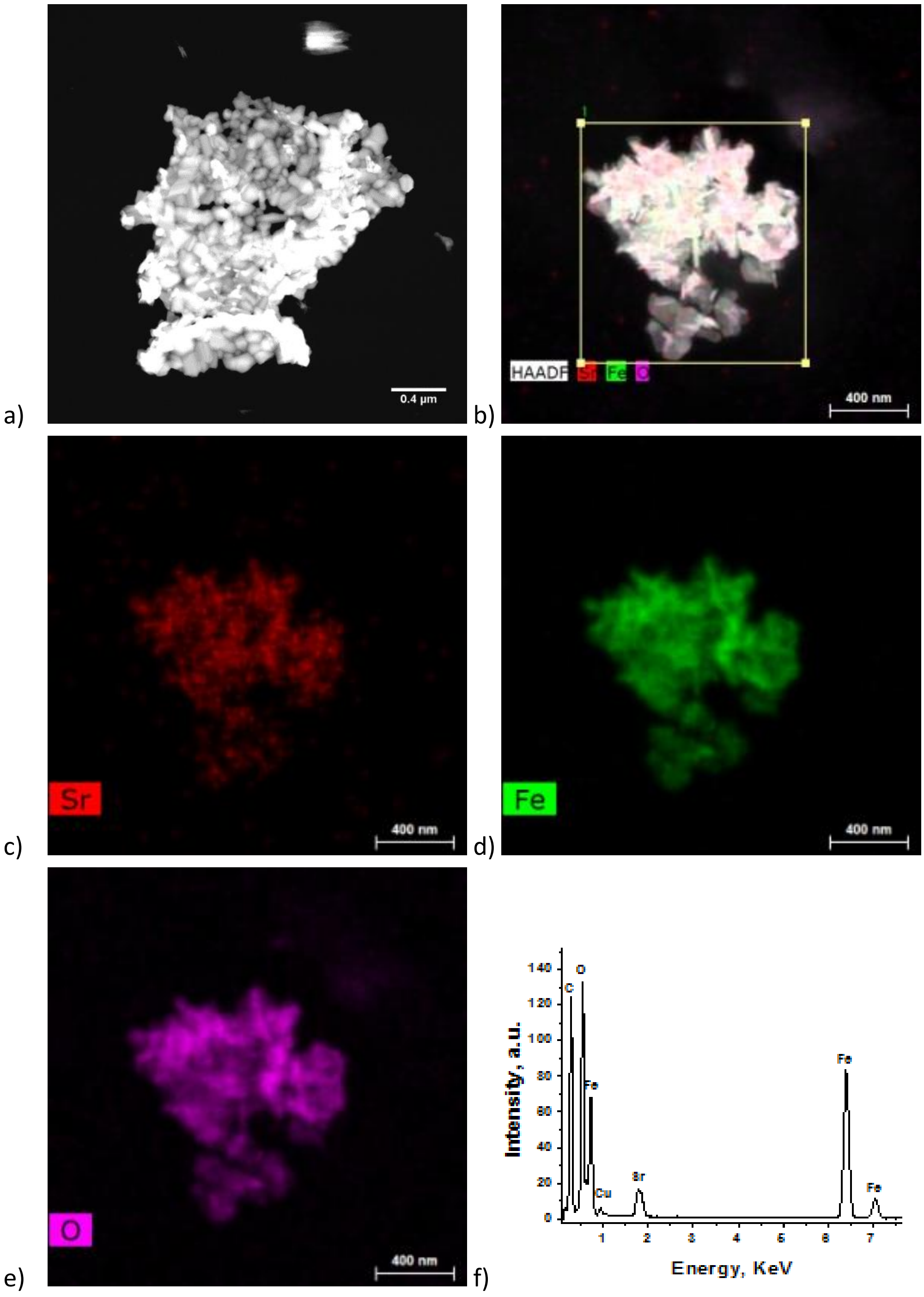

Figure S3a) STEM image of conventional sol-gel synthesised $SrFe_{12}O_{19}$. HAADF (b) and elemental mapping of Sr (c), Fe (d), and O (e). f) is the obtained EDS spectrum.

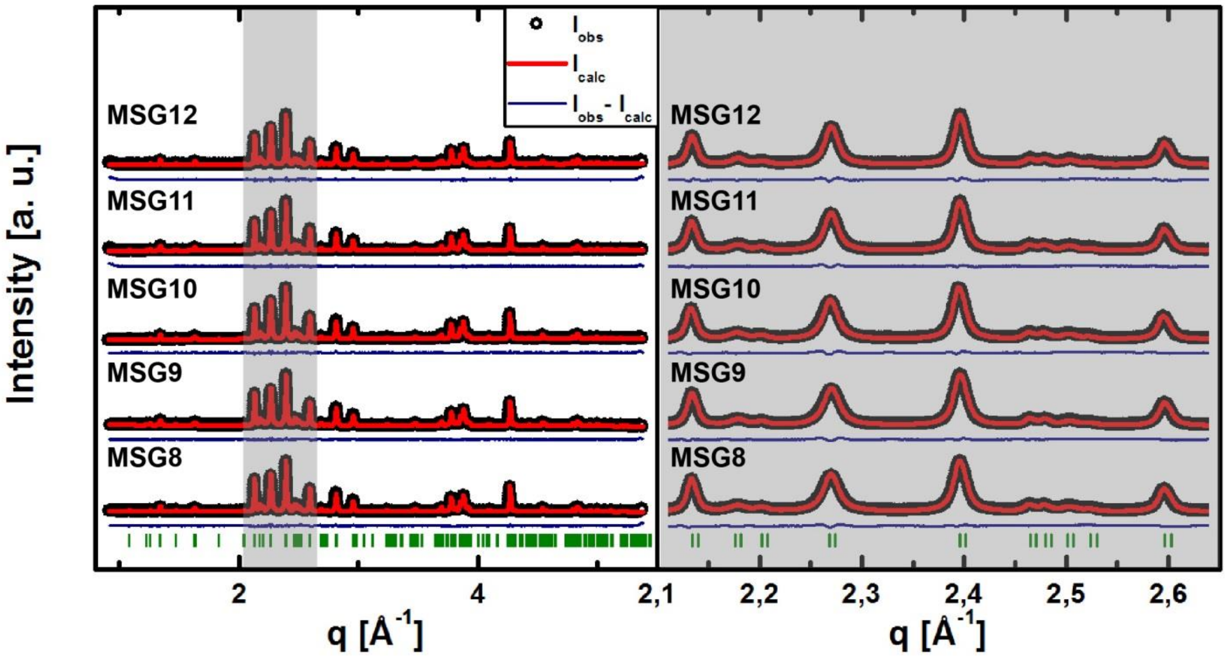

Figure S4 PXRD patterns and Rietveld refinements of $SrFe_{12}O_{19}$ nanocrystallites prepared from different Fe^{3+}:Sr^{2+} molar ratio. To the right a zoom of the data between 2.1 and 2.65 Å^{-1} is shown.

Modified sol-gel synthesis steps:

When the orange-green precursor gel is heated at 450 °C it reacts, and rise resulting in a brown and porous powder with air beneath. When a large amount of gel is held in the crucible and heated, only a part of the powder rise. All the gel become brown, but the lower part of the sample is more like a brown lump rather than porous as the rest. The transition of Fe^{3+} and Sr^{2+} ions to $SrFe_{12}O_{19}$ apparently requires that oxygen is available. If the reacted powder close to the surface shields the gel below from a sufficient amount of oxygen, we hypothesis, that it may turn into α-Fe_2O_3, since the hematite phase forms more readily under deficient oxygen conditions. Sample a) in Figure S5 is made by heating the gel at 450 °C for one hour, hereafter it is calcined at 790°C for one hour. Sample b) is made by heating the gel for five hours at 450 °C resulting in a slight decrease of hematite. The sample shown in Figure S4 c) is heated at 450 °C for one hour and afterwards calcined at 790°C. Here, the sample is agitated twice during the first heating. The last sample shown in d) is prepared by distributing the gel in a thin layer to ensure a homogeneous O2 availability throughout the gel. Consequently, the final product contains 2.8(1) wt% α-Fe_2O_3. All samples are made from the same precursor batch.

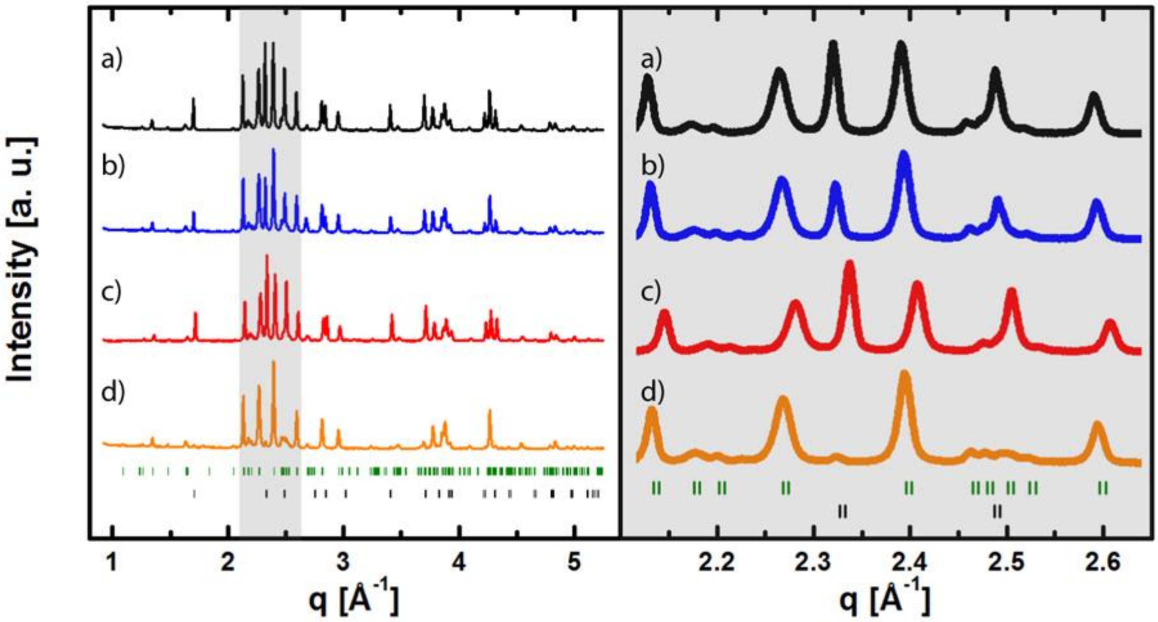

Figure S5 Powder samples made from the same batch prepared under different oxygen conditions. a) gel is heated at 450 °C for one hour and afterwards 790 °C for one hour. b) is heated at 450 °C for five hours and calcined at 790 °C for one hour. c) is heated under equal conditions as a) but agitated twice during the first heating. d) is heated at equal conditions as a) but the gel is distributed in a very thin layer resulting in a significant lower amount of α-Fe_2O_3.

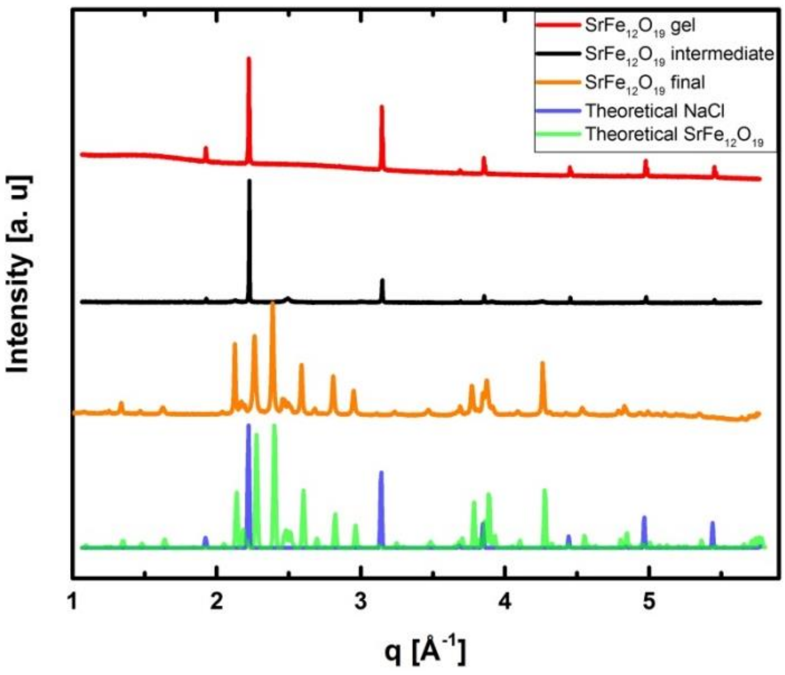

Figure S6 PXRD patterns of MSG9. Red is the PXRD pattern of the dried gel, black is the pattern after the gel has been heated at 450 °C for 1 hour, orange is the diffraction pattern of the final $SrFe_{12}O_{19}$ product, and the green and blue lines are the theoretical PXRD patterns of $SrFe_{12}O_{19}$ and NaCl, respectively.

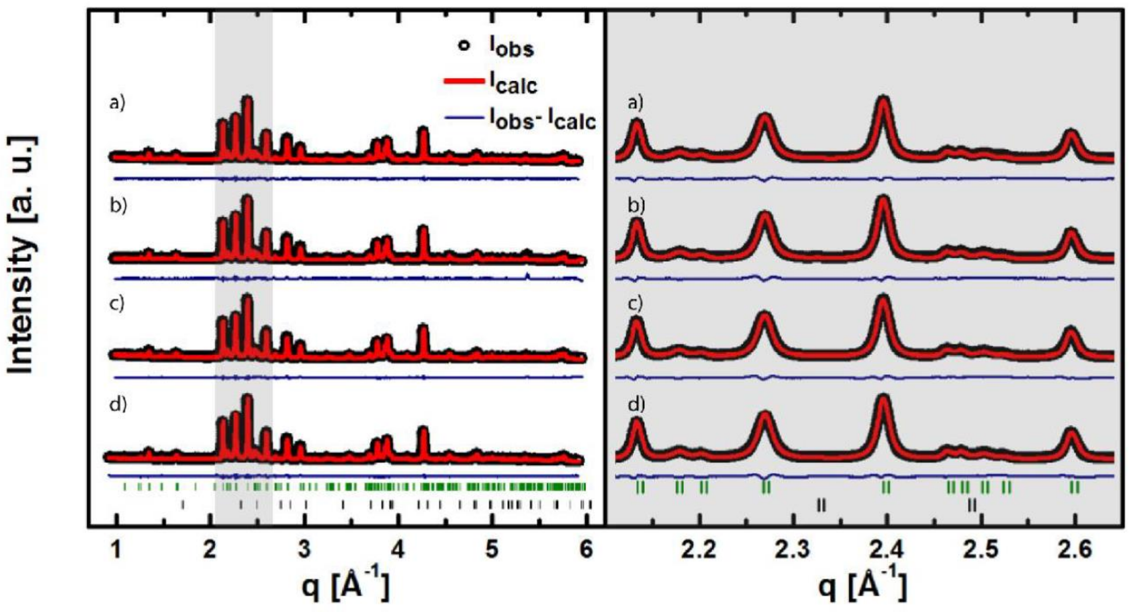

Figure S7: The Rietveld refinement of the PXRD data of SrFe12O19 synthesised with different excess of NaCl. a) has 50% excess of NaCl, b) 100%, c) 200% and d) 300%. To the right a zoom of the data between 2.1 and 2.65 Å-1 is shown.

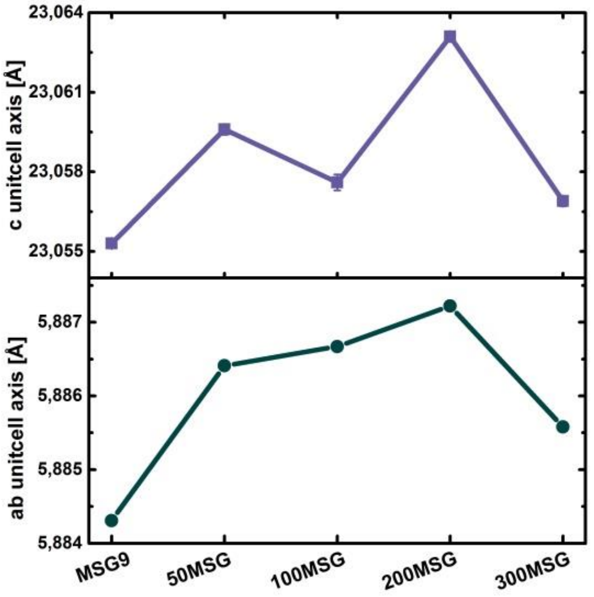

Figure S8 Values of the unit cell axes of MSG synthesised samples prepared with 50-300 % added NaCl relative to the amount of formed NaCl in the parent synthesis. The uncertainties are, unless shown, smaller than the symbols.

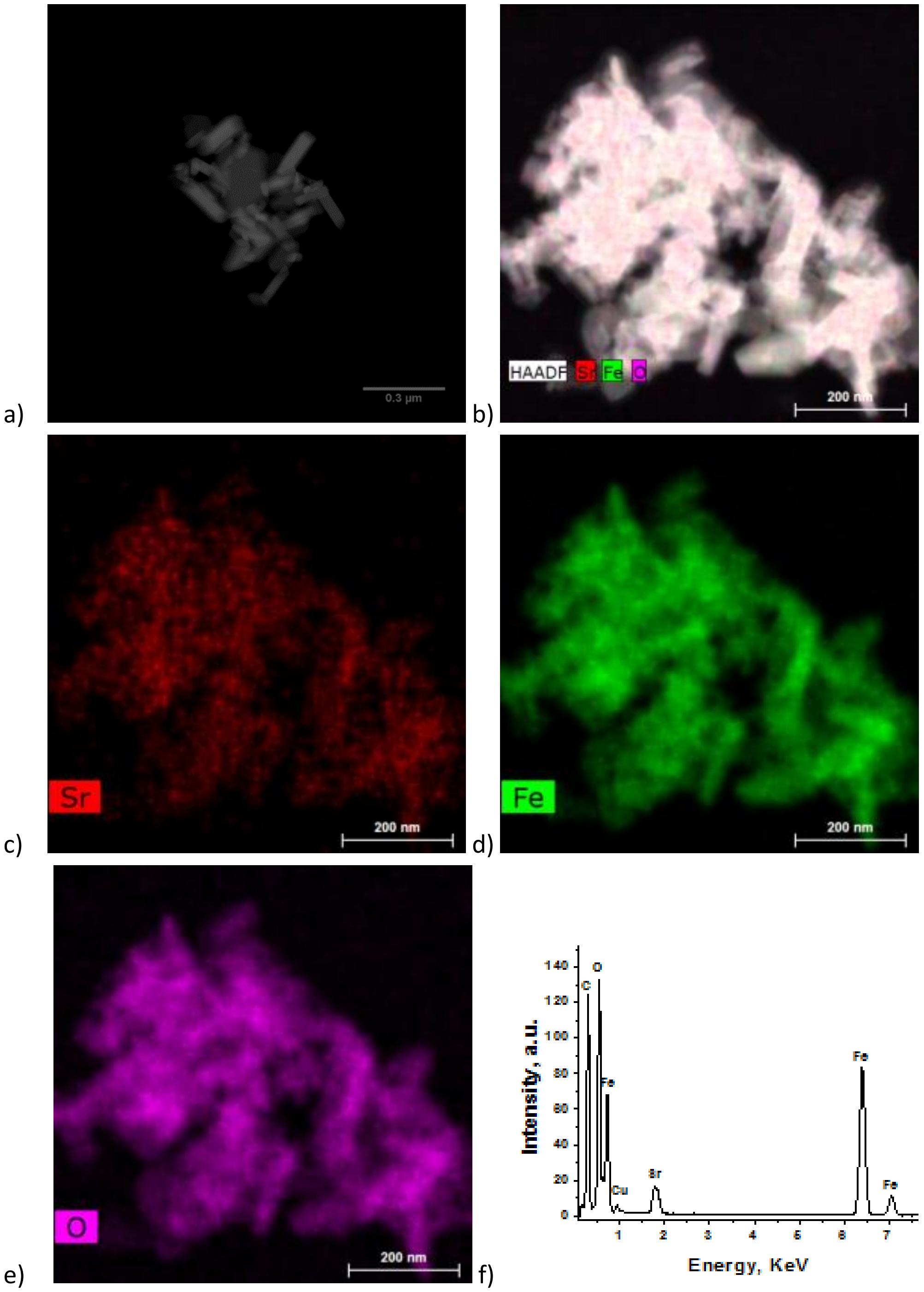

Figure S9a) STEM image of modified sol-gel synthesised $SrFe_{12}O_{19}$. HAADF (b) and elemental mapping of Sr (c), Fe (d), and O (e). f) is the obtained EDS spectrum.

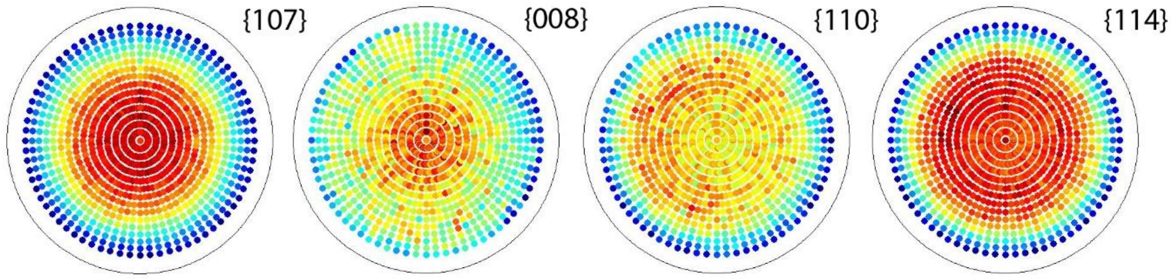

Figure S10 Pole figure measurement of SPS_CSG

a)

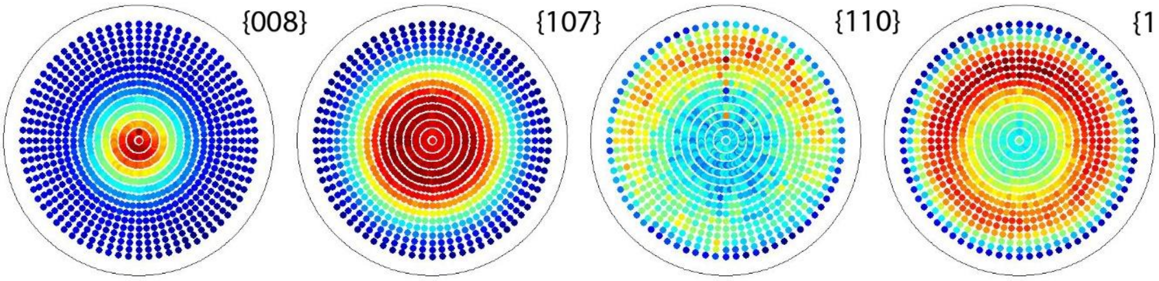

b)

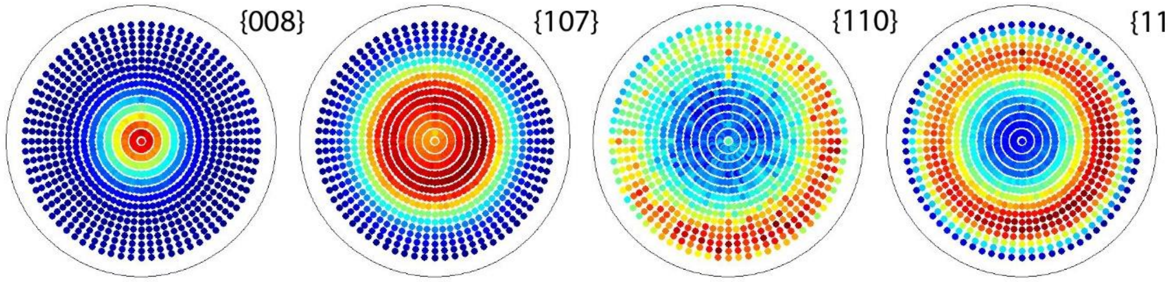

c)

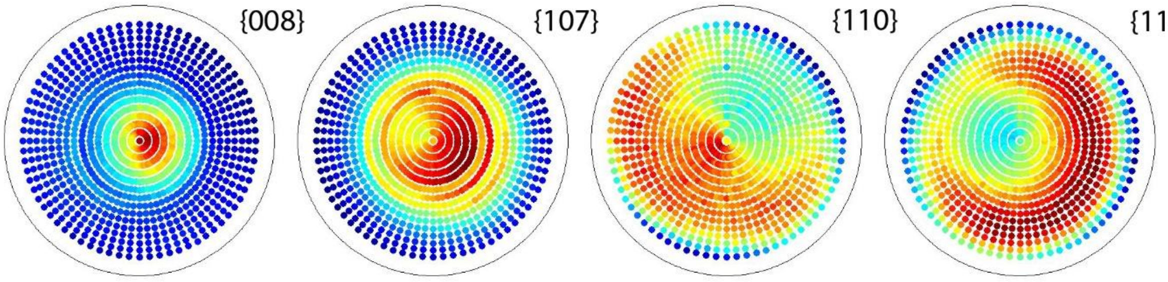

Figure S11 Pole figure measurement of a) SPS_MSG750, b) SPS_MSG800, and c) SPS_MSG850. The differences in intensity throughout the circle is due to imperfect alignment but do not affect the extracted data.
